# Awareness of Non-alcoholic Fatty Liver Disease and Its Determinants in Jazan, Saudi Arabia: A Cross-Sectional Study

**DOI:** 10.7759/cureus.53111

**Published:** 2024-01-28

**Authors:** Alfadl A Abdulfattah, Erwa Eltayib Elmakki, Bushra I Maashi, Bushra A Alfaifi, Asmaa S Almalki, Njoud AL Alhadi, Hamad Majrabi, Abdulrahman Kulaybi, Ayoub Salami, Fouad I Hakami

**Affiliations:** 1 Internal Medicine, Jazan University, Jazan, SAU; 2 College of Medicine, Jazan University, Jazan, SAU; 3 Clinical Nutrition, Jazan University, Jazan, SAU; 4 Emergency Department, King Fahad Central Hospital, Jazan, SAU

**Keywords:** jazan, awareness, middle east, liver disease, nafld

## Abstract

Background

Non-alcoholic fatty liver disease (NAFLD) is characterized by hepatic steatosis. It is the leading cause of liver-related mortality, end-stage liver disease, and the need for liver transplantation. This study aimed to assess the level of awareness regarding NAFLD among the adult population in Jazan, Saudi Arabia, and to explore the determinants of the awareness level.

Method

This descriptive cross-sectional study was conducted among the general population of Jazan, Saudi Arabia, under the supervision of Jazan University, excluding those with end-stage liver failure. An online self-administered questionnaire, adopted from the literature, was sent through different social media platforms. A total of 1,034 people participated in this study. The chi-square test and multiple linear regression were used to identify the predictors of NAFLD awareness.

Results

Fifty-three percent of the participants were aware of NAFLD. The mean score of the participants' awareness was 22.7 ± 4.9 out of 40 points. Employed (95% confidence interval (CI): -1.9, -0.03; p = 0.044) and private business individuals (95% CI: -3.5, -1.1; p < 0.001) had a lower awareness level than students. The higher income level was associated with greater awareness (95% CI: 0.36, 2.4; p = 0.008). Those who had never heard of NAFLD had nearly twice as much awareness (95% CI: -3.0, -0.67; p = 0.002).

Conclusions

Nearly half the study participants displayed good awareness of NAFLD. However, efforts must focus on awareness campaigns, encouraging health-seeking behaviors, and targeted screening for early detection and treatment, especially in at-risk populations, since many participants were unaware of NAFLD.

## Introduction

Non-alcoholic fatty liver disease (NAFLD) represents a complex spectrum of liver disorders characterized by hepatic steatosis and excessive fat accumulation within the liver without secondary causes, such as heavy alcohol consumption. This spectrum ranges from non-alcoholic fatty liver (NAFL), which tends to be relatively benign, to a more severe and potentially progressive form known as non-alcoholic steatohepatitis (NASH). Left unaddressed, NAFLD can lead to fibrosis and cirrhosis, which pose significant threats to public health [1,2].

NAFLD is distinctly categorized into two types. The first type is closely associated with metabolic syndrome. The prevailing understanding implicates insulin resistance as the primary underlying mechanism [3-5]. Individuals with NAFLD often exhibit one or more components of metabolic syndrome, such as systemic hypertension, dyslipidemia, insulin resistance, or overt diabetes [6].

NAFLD has surged globally, affecting approximately 30.05% of the world's population [7]. It is the leading cause of liver-related mortality [8] and end-stage liver disease [9], often necessitating liver transplantation [10]. Escalating epidemics of obesity and type-2 diabetes mellitus have fueled the rise in NAFLD [11,12]. The Middle East has witnessed a notable increase in NAFLD prevalence in recent years, from 36.53% in 1990 to 42.62% in 2019 [7]. A systematic review reported an NAFLD prevalence of 16.8% in Saudi Arabia [13]. In particular, the obesity rates in Saudi Arabia exceed the global average, contributing to a higher prevalence of NAFLD in the region [14].

Despite the growing global significance of NAFLD, awareness of this condition remains dismally low. In the United States, NAFLD awareness is only 4.4%, representing a glaring discrepancy compared to the awareness levels of other liver-related disorders, such as viral hepatitis [15]. European countries exhibited similarly low awareness rates [16]. One study found that only 2.4% of the individuals diagnosed with NAFLD were aware of their condition [17].

However, the importance of raising awareness of NAFLD cannot be overstated. Elevated awareness is a pivotal step toward increasing treatment uptake, facilitating informed decisions, and promoting crucial behavioral changes, such as lifestyle modifications. Beyond its clinical implications, NAFLD has a significant economic impact and diminishes the overall health-related quality of life of affected individuals [18].

This study aimed to assess the level of NAFLD awareness among the adult population in Jazan, Saudi Arabia, and to identify the factors influencing this awareness.

## Materials and methods

This study represents a descriptive cross-sectional investigation undertaken within the geographic confines of the Jazan region, situated in the southwestern expanse of Saudi Arabia, contiguous with the Red Sea, encompassing an approximate population of 1.5 million residents. The study included the population of Jazan aged 18 years or above who possessed basic literacy skills and had social media platforms. Individuals with end-stage liver failure were excluded.

The sample size was determined using the following formula:

n = z²p(1-p)/d²,

where *Z* corresponds to a 95% confidence level, *p* denotes the anticipated population proportion, and *d *denotes the 5% margin of error. In addition, a 10% nonresponse rate was factored in, yielding a minimum sample size of 440 participants.

Data collection

A preliminary pilot study involving a selected group of participants was conducted to assess the questionnaire's clarity and understanding. Participant feedback from the preliminary phase was incorporated into the final survey. The survey tool was disseminated to participants through Google Forms, Facebook, and WhatsApp, employing convenience sampling.

The questionnaire comprised two principal sections. The first segment collected demographic data, including age, gender, nationality, employment status, educational qualifications, and monthly income. The second section featured the validated questionnaire derived from previous studies [19] that explored the participants’ NAFLD knowledge, including prior liver disease diagnoses among respondents or their close relatives. Moreover, it delved into the participants' awareness of NAFLD, including their comprehension of the risk factors, diagnostic methodologies, treatment modalities, associated symptoms, and potential complications arising from this condition. Furthermore, the participants' perceptions toward NAFLD curability, preventability, and potentially life-threatening effects were assessed.

A structured scoring system was implemented to assess NAFLD awareness. Correct, incorrect, and neutral responses were assigned scores of 2, 0, and 1, respectively. Subsequently, a cumulative awareness score was computed for each participant, resulting in scores ranging from 0 to 40 points. Higher scores indicated greater levels of awareness.

The normality of the data distribution was examined using the Kolmogorov-Smirnov test. To categorize awareness data, the median score was employed as a delineating threshold, bifurcating participants into those classified as "aware" (those scoring greater than or equal to the median) and those designated as "not aware" (those scoring less than the median). Categorical data were presented using frequency and percentage. To identify the factors associated with NAFLD awareness, multiple linear regression analysis was performed. The statistical significance of the study was set at p < 0.05.

Ethical considerations 

Ethical approval was obtained from the Jazan Health Ethics Committee (IRB No.: REC-44/05/404). Each participant was provided with a comprehensive explanation of the study's objectives. Written informed consent was obtained from all participants, affirming their voluntary participation in the study. The ethical preference for participant withdrawal at any point was emphasized. The anonymity and confidentiality of participants' identities and responses were maintained.

## Results

In this study, 1,034 participants were enrolled, primarily within the 18-24-year age group (48.5%). Most participants were female (72.9%), of Saudi nationality (96.7%), and possessed a bachelor's degree (61.1%). The predominant occupational category was students (38.4%), with the majority reporting a monthly income below 5000 Saudi Riyals (58.7%). Notably, a substantial proportion of participants (62.3%) indicated no prior awareness of NAFLD, with 14.3% reporting educational curricula as their primary source of information on NAFLD (Table 1).

**Table 1:** Sociodemographic characteristics of the study participants SR: Saudi Riyals

Characteristic	n	(%)
Age (years)		
18-24	502	48.5%
25-34	283	27.4%
35-44	162	15.7%
45-54	69	6.7%
55-64	15	1.5%
65 and above	3	0.3%
Gender		
Female	754	72.9%
Male	280	27.1%
Nationality		
Non-Saudi	34	3.3%
Saudi	1,000	96.7%
Level of education		
Primary	6	0.6%
Intermediate	25	2.4%
Secondary	232	22.4%
Bachelor's	632	61.1%
Others	139	13.4%
Occupation		
Employed	239	23.1%
Private business	79	7.6%
Housewife	116	11.2%
Student	397	38.4%
Retired	203	19.6%
Income level		
Less than 5000 SR	607	58.7%
5000-10,000 SR	222	21.5%
10,000-15,000 SR	135	13.1%
More than 15,000 SR	70	6.8%
Source of information		
Friends/relatives	55	5.3%
From my curriculum	148	14.3%
From my doctor	31	3%
Social media	99	9.6%
Others	57	5.5%
I have never heard about it before	644	62.3%

It was ascertained that 9.4% of the participants had either a personal diagnosis of NAFLD or were acquainted with it through a relative’s diagnosis. Notably, 77% of the respondents acknowledged NAFLD's potential to induce hepatic inflammation, whereas 50% were ignorant regarding its presenting symptoms. In addition, 47% identified obesity as a prevalent risk factor, with 39% recognizing end-stage liver disease as a potential complication. Of the participants, 42% perceived NAFLD as preventable, 50% believed it was curable, and 53% considered it a life-threatening condition (Table 2).

**Table 2:** Awareness regarding NAFLD among the adult population of Jazan NAFLD: non-alcoholic fatty liver disease, BMI: body mass index, CT: computed tomography, MR: magnetic resonance, USG: ultrasound sonography

Characteristic	N = 1,034^1^
Have you or someone you know ever been diagnosed with NAFLD?	
Yes*	97 (9.4%)
No	732 (71%)
I don’t remember	205 (20%)
NAFLD is characterized by the accumulation of………	
Fats in heart	8 (0.8%)
Fats in the liver*	639 (62%)
I don’t know	359 (35%)
Oil in liver	28 (2.7%)
Do you think NAFLD can only occur if it runs in the family?	
Agree	174 (17%)
Disagree*	352 (34%)
I don’t know	508 (49%)
Do you think healthy eating habits can prevent NAFLD?	
Agree*	708 (68%)
Disagree	76 (7.4%)
I don’t know	250 (24%)
People with low blood pressure are at higher risk of NAFLD.	
Agree	244 (24%)
Disagree*	151 (15%)
I don’t know	639 (62%)
Diabetes is related to NAFLD	
Agree*	362 (35%)
Disagree	131 (13%)
I don’t know	541 (52%)
Only adults are prone to the development of NAFLD.	
Agree	291 (28%)
Disagree*	208 (20%)
I don’t know	535 (52%)
How many stages can occur in NAFLD disease?	
2	70 (6.8%)
3	171 (17%)
4*	119 (12%)
I don’t know	674 (65%)
What is the most commonly presented symptom of NAFLD?	
Fatigue and lethargy*	194 (19%)
Headache	24 (2.3%)
Nausea and vomiting	297 (29%)
I don’t know	519 (50%)
Which methods are helpful for doctors to diagnose NAFLD?	
Blood tests	63 (6.1%)
BMI	45 (4.4%)
Imaging techniques	94 (9.1%)
All of these*	477 (46%)
I don’t know	355 (34%)
What is the standard method for definite diagnosis of NAFLD?	
CT	70 (6.8%)
Liver biopsy*	330 (32%)
MR	75 (7.3%)
USG	47 (4.5%)
I don’t know	512 (50%)
What is the standard method for definite treatment of NAFLD?	
Angioplasty	50 (4.8%)
Laparoscopy	127 (12%)
Surgical intervention	157 (15%)
None*	81 (7.8%)
I don’t know	619 (60%)
NAFLD can cause... in the liver	
Inflammation*	795 (77%)
Injury	341 (33%)
Swelling	691 (67%)
What can be the associated complications of NAFLD?	
Liver cancer*	106 (10%)
End-stage liver disease*	406 (39%)
Brain tumor	15 (1.5%)
Cardiovascular disease	73 (7.1%)
Skeletal dysfunction	12 (1.2%)
I don’t know	422 (41%)
What are the common risk factors associated with NAFLD?	
Obesity*	483 (47%)
Metabolic disorders*	416 (40%)
Diabetes mellitus*	316 (31%)
Parkinson’s disease	111 (11%)
Dermatitis	117 (11%)
I don’t know	460 (44%)
Do you think NAFLD can be prevented?	
Yes*	431 (42%)
No	94 (9.1%)
I don’t know	509 (49%)
Do you think NAFLD can be cured?	
Yes*	521 (50%)
No	85 (8.2%)
I don’t know	428 (41%)
Can NAFLD be life-threatening?	
Yes*	551 (53%)
No	72 (7.0%)
I don’t know	411 (40%)
^1^n (%), *correct answer	

**Figure 1:**
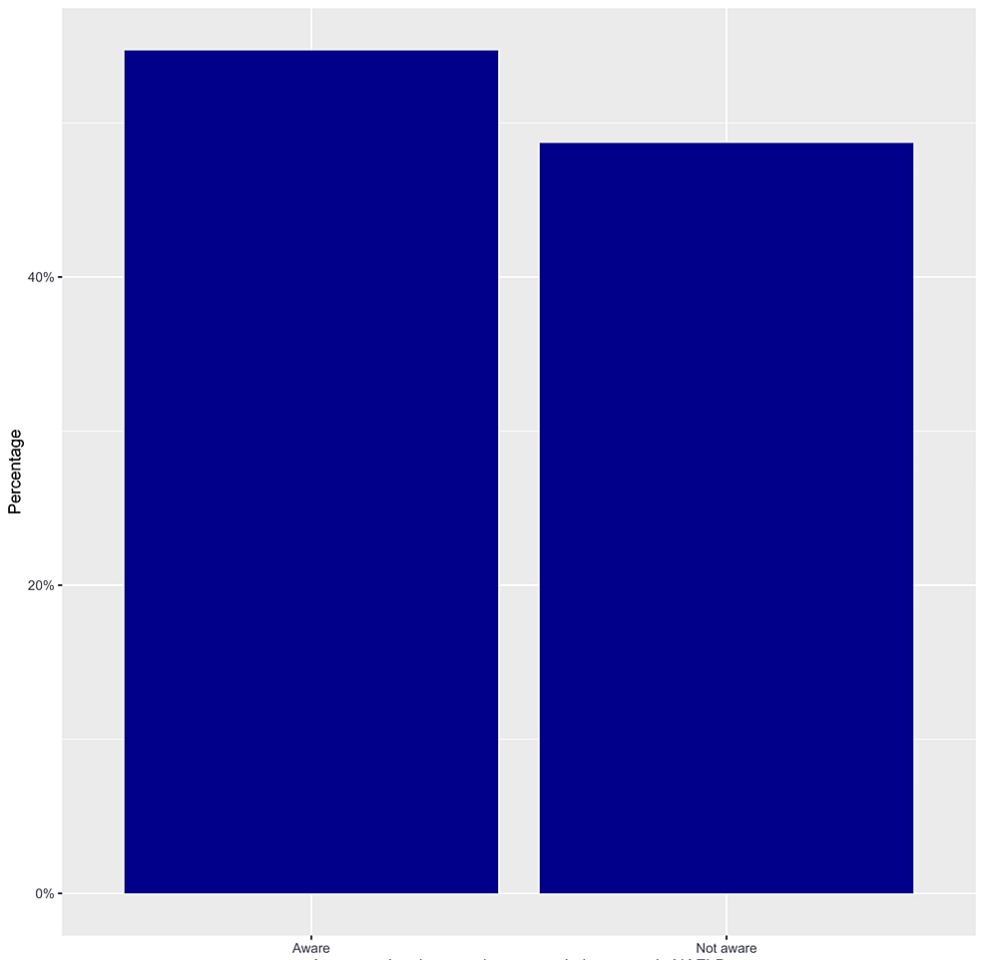
Awareness level of NAFLD among the adult population of Jazan

The mean awareness score was 22.7 ± 4.9 out of a maximum attainable score of 40, with a median score of 22. Notably, 52.9% of the participants were categorized as "aware" of NAFLD, while 47.1% fell into the "not aware" category (Figure 1).

Distinct patterns emerged when assessing the determinants of NAFLD awareness among the study participants. Employed individuals exhibited a 0.97-point decrease in awareness levels compared to students (95% CI: -1.9, -0.03, p = 0.044), while participants engaged in private business demonstrated a substantially greater decrement, with a 2.3-fold reduction in awareness compared to students (95% CI: -3.5, -1.1; p < 0.001). Furthermore, participants within the income bracket of 10,000-15,000 Saudi Riyals exhibited 1.4 times higher awareness than those with incomes below 5,000 Saudi Riyals (95% CI:0.36, 2.4; p = 0.008). Notably, participants with no prior exposure to information about NAFLD were found to possess nearly half the level of awareness as those who had received information from friends or relatives (95% CI: -3.0, -0.67; p = 0.002) (Table 3).

**Table 3:** Predictors of NAFLD awareness among the adult population of Jazan ^1^CI: confidence interval; SR: Saudi Riyals; NAFLD: non-alcoholic fatty liver disease

Characteristic	Beta	95% CI^1^	p value
Nationality			
Non-Saudi	—	—	
Saudi	1.1	-0.57, 2.7	0.2
Occupation			
Student	—	—	
Employed	-0.97	-1.9, -0.03	0.044
Housewife	-0.20	-1.1, 0.74	0.7
Private business	-2.3	-3.5, -1.1	<0.001
Retired	-0.72	-1.5, 0.10	0.086
Income level			
Less than 5000 SR	—	—	
5000-10,000 SR	0.11	-0.72, 0.94	0.8
10,000-15,000 SR	1.4	0.36, 2.4	0.008
More than 15,000 SR	0.21	-0.99, 1.4	0.7
Source of information			
Friends/relatives	—	—	
I have not heard about it before	-1.8	-3.0, -0.67	0.002
Social media	-0.77	-2.2, 0.63	0.3
Others	0.00	-1.9, 1.9	>0.9

Being Saudi or a student and having a bachelor's degree was associated with higher NAFLD awareness (p < 0.05). An income of less than 5000 Saudi riyals was associated with higher NAFLD awareness (p < 0.05) (Table 4).

**Table 4:** Relationship between the participants’ sociodemographic characteristics and their awareness level ^1^n (%); ^2^Pearson's chi-squared test; Fisher's exact test; SR: Saudi Riyals

	Awareness level		
Characteristic	Aware, N = 547^1^	Not aware, N = 487^1^	p value^2^
Gender			0.089
Female	411 (75%)	343 (70%)	
Male	136 (25%)	144 (30%)	
Age (years)			0.2
18-24	278 (51%)	224 (46%)	
25-34	148 (27%)	135 (28%)	
35-44	82 (15%)	80 (16%)	
45-54	28 (5.1%)	41 (8.4%)	
55-64	10 (1.8%)	5 (1.0%)	
65 and above	1 (0.2%)	2 (0.4%)	
Nationality			0.005
Non-Saudi	10 (1.8%)	24 (4.9%)	
Saudi	537 (98%)	463 (95%)	
Level of education			<0.001
Primary	1 (0.2%)	5 (1.0%)	
Intermediate	11 (2.0%)	14 (2.9%)	
Secondary	100 (18%)	132 (27%)	
Bachelors	380 (69%)	252 (52%)	
Others	55 (10%)	84 (17%)	
Occupation			<0.001
Employed	126 (23%)	113 (23%)	
Housewife	59 (11%)	57 (12%)	
Private business	30 (5.5%)	49 (10%)	
Student	239 (44%)	158 (32%)	
Retired	93 (17%)	110 (23%)	
Income level			0.024
Less than 5000 SR	328 (60%)	279 (57%)	
5000-10,000 SR	101 (18%)	121 (25%)	
10,000-15,000 SR	83 (15%)	52 (11%)	
More than 15,000 SR	35 (6.4%)	35 (7.2%)	
Source of information			<0.001
From my curriculum	128 (23%)	20 (4.1%)	
Friends/relatives	30 (5.5%)	25 (5.1%)	
I have not heard about it before	282 (52%)	362 (74%)	
Others	54 (9.9%)	34 (7.0%)	
Social media	53 (9.7%)	46 (9.4%)	

## Discussion

NAFLD is a common benign liver disease. However, it can progress to fibrosis in approximately 32% of cases and to cirrhosis in 20% of cases, over a 10-year course [20]. Despite the high prevalence of NAFLD in Saudi Arabia, studies on the awareness of this disease among the general population are few.

Therefore, the current study aimed to assess the awareness of NAFLD among the population of Jazan. Employed and private business workers were significantly more aware of NAFLD than those who belonged to other occupations (p = 0.044 and 0.001, respectively).

There was a significant correlation between the awareness level and Saudi nationality (p = 0.005). This could be attributed to the high socioeconomic levels and educational privileges among the original Saudi residents compared to non-Saudis who are mostly marginalized or illiterate workers.

The sources of information for most participants were their curriculum (14.3%) and social media (9.6%), which are similar to those of a study on Mexican women where their information sources on NAFLD were mostly family and media [21].

In the present study, 53% of the respondents were aware of NAFLD. By contrast, a similar study by Aljahdli et al. in 2021 on the Saudi population in Jeddah reported an overall awareness of 33% among the study participants [22]. This difference could be attributed to differences between the study samples, improvements in health education programs, and the growing use of smartphones and social communication platforms in Saudi Arabia. 

In the current study, 71% of the participants reported that they or their relatives had never been diagnosed with NAFLD, and approximately 48% had a low level of awareness about the disease. Another Asian community-based study conducted in 2020 in Singapore revealed that 71.2% of the participants had heard about NAFLD; a more recent study reported awareness at 75% [23,24].

Despite both being Asian countries and developed economies, the Singaporean population had a better awareness of NAFLD than the Saudi population. Both communities appreciate the fact that NAFLD can be dangerous and life-threatening (53% in Saudi Arabia and 68.8% in Singapore) [23].

Nevertheless, most of our study participants indicated that the disease could be prevented (42%) and cured (50%), compared with 2% and 1%, respectively, in the Brooklyn population [20]. When asked about the familial nature of the disease, 17% of our participants agreed that NAFLD runs in the family, compared to 70% of the United States population who indicated that it is hereditary. A study from a different continent concluded that approximately 84% of the United States population was unaware of NAFLD and the conditions that could predispose them to it. When asked about the risk factors for NAFLD, 44% of our participants indicated their ignorance, compared to 83% of the Brooklyn-USA residents [20]. Interestingly, 49% of the South Korean population was aware of NAFLD, and less than half were aware of its risk factors [25].

Limitations and strengths of the current study

The present study provides significant findings regarding the awareness of NAFLD and its risk factors among people in the Jazan region of Saudi Arabia. However, it also has some limitations. One of the main drawbacks of this study is the use of an online self-administered questionnaire, which may result in a high frequency of missed data and inaccurate answers compared to an interview-based questionnaire. Moreover, only those with good Internet access could have been included in this study. Second, most study respondents and participants were young students, causing an indirect selection bias that could have affected the conclusion. We hope that these limitations will be addressed in future survey-based observational studies.

The strengths of this study include the use of a validated Arabic-translated questionnaire. Furthermore, the sample was adequate and representative of the entire population and the results and conclusions represent an excellent addition to the medical literature. Finally, the present study will undoubtedly enrich the epidemiological data regarding the awareness of NAFLD and its risk factors among the Saudi population. 

Recommendations

Based on the outcomes of this study, we recommend strategic planning of awareness campaigns about NAFLD among residents of Jazan and other Saudi areas, in addition to conducting more nationwide surveys to establish awareness levels among the Saudi population and assess their health-seeking behaviors.

## Conclusions

More than half of the study participants have good knowledge about NAFLD, yet around 47.1% have low awareness levels, which necessitate establishing awareness programs and enhancing health-seeking behaviors in addition to screening at-risk populations for early diagnosis and treatment of this prevalent disease. The predictors of awareness among our participants were private businesses and high income level. There were no differences in the awareness level between males and females and between the different age groups. Nevertheless, there was a statistically significant difference in awareness level according to nationality, educational level, occupation, and level of income.
